# Machine learning in population health: Opportunities and threats

**DOI:** 10.1371/journal.pmed.1002702

**Published:** 2018-11-27

**Authors:** Abraham D. Flaxman, Theo Vos

**Affiliations:** Health Metrics Sciences, University of Washington, Seattle, Washington, United States of America

## Abstract

Abraham D. Flaxman and Theo Vos of the Institute for Health Metrics and Evaluation, University of Washington, discuss near-term applications for ML in population health and name their priorities for ongoing ML development.

Machine learning (ML) has succeeded in complex tasks by trading experts and programmers for data and nonparametric statistical models. However, the applications for which ML has been successfully deployed in health and biomedicine remain limited [1]. These limits also apply in population health, in which we are concerned with the health outcomes of a group of individuals and the distribution of outcomes within the group. In our metrics, we deal with messy global health data, and a large effort goes into piecing together sparse, noisy information to understand what causes how much health loss, where it occurs, and how it is changing. In our interventions, we often face stringent constraints on resources and need to develop appropriate and acceptable solutions under these constraints. How might ML-based approaches change population health? Here, we discuss opportunities and threats from ML, with our views on further development needed within ML to create the best possible outcomes.

As a start, ML and artificial intelligence (AI) can automate tasks that people do not like doing, cannot do fast enough, or cannot afford to do. AI luminary Andrew Ng provides this concise guidance: “[i]f a typical person can do a mental task with less than one second of thought, we can probably automate it using AI either now or in the near future” [1]. Following Ng’s heuristic, the implementation challenge is breaking these tasks down into pieces that a person could do in less than a second. For example, our process of vetting results in the Global Burden of Disease Study [2] included the visual inspection of thousands of plots showing data together with model estimates. Individual researchers are unlikely to notice and follow up on all abnormal plots. Our own preliminary work suggests that a convolutional neural network can accurately screen such plots and pass on the few hundreds that are suspicious for a human to review. Open-source ML software like Scikit-Learn and Keras facilitates this, but operational research into how best to apply existing methods could drive wider adoption. ML approaches are not easy to develop or deploy, and we still lack a sufficient range of experience and case studies to know when an ML solution will be worth the effort.

Another promising example of ML-based automation comes from the challenge of mapping the results of verbal autopsy interviews to the underlying cause of death. Cause-specific death data are an important component of disease burden estimation, but globally, nearly two out of three deaths go unrecorded. The verbal autopsy is a structured interview that can provide some information to fill this gap, but the process of mapping from the interview results to the underlying cause has traditionally required a doctor with experience in the location where the death occurred. These experts are in short supply, and verbal autopsy efforts can end up with multiyear delays between collecting data and mapping them to the underlying cause. ML methods for computer certification of verbal autopsy can provide accuracy similar to expert humans, without the delay [3].

## Fairness, accountability, and transparency

Understanding why ML methods predict as they do is a relatively new area of research. This is often referred to as fairness, accountability, and transparency in ML (FAT/ML) or Explainable AI and is a focus area of another perspective in this collection [4]. Although the parallel terminology connects to slightly different foci of these lines of research, both address a potential weakness of many current ML methods, which is the inability of the researcher to explain why the machine has predicted as it has. Work in clinical medicine has identified the importance of explainable prediction methods [5]. We believe, for population health as well, a mechanism for explaining ML-based predictions will increase opportunities for deploying ML methods—uptake will increase if there is an intuitive explanation or demonstration that a method has followed a plausible pattern. In our Global Burden of Disease work, an objection to policy implications derived from complex modeling exercises is that they cannot be trusted. The Guidelines for Accurate and Transparent Health Estimates Reporting (GATHER) were developed to address objections like this and to facilitate model explanations in scholarly communication [6]. Our experience developing methods for computer certification of verbal autopsy has bolstered our belief that using an explainable approach, even with a reduction in accuracy, can be superior. In verbal autopsy, we have recommended a simpler approach (Tariff) over a complex ML method (random forest) [3], and this has aided in subsequent survey design [7] and seems to have facilitated adoption by public health practitioners.

## Privacy-preserving ML methods

Formal definitions and guarantees of privacy have emerged recently from work at the intersection of cryptography, statistics, and computer security [8]. Methods that provide data-driven insights without leaking data secrets could be useful in population health, for which valuable data are often not shared, because of privacy concerns. Privacy-preserving ML methods could provide a technological opportunity to glean insights from large, private datasets. However, when developing this line of inquiry specifically for applications in population health, researchers should consider the multiple potential reasons that datasets are not released publicly. Some, such as ethical and regulatory requirements, may be addressed by technologies like differential privacy, whereas others, such as misaligned strategic incentives among researchers, might require social as well as technical innovation to remedy. To make this concrete, consider the GATHER guidelines, which allow “[f]or any data inputs that cannot be shared because of ethical or legal reasons, such as third-party ownership, [to] provide a contact name or the name of the institution that retains the right to the data” [6]. A technical solution that permitted limited sharing of data inputs would promote reproducibility more directly than contact information.

## Causal inference

The weaknesses that many ML applications have with explanation also relate to a weakness in making claims about causation. However, the central question underlying many population health inquiries is about just such causal claims. If we scale up a health program, introduce a new vaccine, or make a change to a health incentive, how will this change population health? Further development and translation of ML methods to go beyond predicting whether a digital image contains a cat to predicting policy outcomes will be of great value. Preliminary work by Kleinberg and colleagues has provided some insightful examples of when predicting causal effects is required [9], and some methods for this purpose are beginning to emerge [10,11].

## Anticipating deleterious effects

Finally, we must anticipate the potential ill effects of ML-enabled technologies on population health and prepare countermeasures. What negative effects of ML should we anticipate? Although numerous science fiction novels can be developed in response, a challenge to population health that is already emerging and being documented occurs when AI is brought to bear on individual-level decisions for social programs [12,13]. This opportunity to streamline underresourced efforts to deliver health and other social services is also a threat, and research into countermeasures against the potential for algorithms to reinforce social inequities may be of great importance to population health.

## Conclusions

ML has reached a point at which it is possible to automate tasks that, until recently, could not be done without substantial human labor. This affords an opportunity in population health for doing more, faster, better, and cheaper, but it is not without risks. Further developments in how to deploy ML methods—especially methods that are explainable, that respect privacy, and that make accurate causal inferences—will help us take advantage of this opportunity.

## Abbreviations

AI: artificial intelligence
FAT/ML: fairness, accountability, and transparency in ML
GATHER: Guidelines for Accurate and Transparent Health Estimates Reporting
ML: machine learning
